# Photostabilities and anti-tumor effects of curcumin and curcumin-loaded polydopamine nanoparticles†

**DOI:** 10.1039/d4ra01246a

**Published:** 2024-04-25

**Authors:** Shufeng Yan, Xiaoyun Liao, Qi Xiao, Qingqing Huang, Xiaochen Huang

**Affiliations:** a Medical Plant Exploitation and Utilization Engineering Research Center, Sanming University Sanming Fujian 365004 China ysfready@fjsmu.edu.cn; b State Key Laboratory of Structural Chemistry, Fujian Institute of Research on the Structure of Matter, Chinese Academy of Sciences Fuzhou Fujian 350002 China

## Abstract

Currently, the photostability of photosensitizer curcumin is the main bottleneck limiting their application, reducing the bioavailability of curcumin. Studying the effect of different light sources on the photostabilities of curcumin and loading it onto polydopamine nanocarriers with better biocompatibility will help improve its light utilization efficiency. In this study, we investigated the photostabilities of curcumin and a polydopamine-based nanoparticle (polydopamine-curcumin composite nanoparticles, PDA-Cur NPs) loaded with curcumin through *in vitro* and *in vivo* experiments to achieve better antitumor effects. The results demonstrated that curcumin has good photostability in dark, but with significant photodegradation rates in both red and blue light. Blue light has a faster effect on the photodegradation of curcumin, with a degradation rate of 42.1% after 10 minutes, which is about 1.7 times that of the red light. Our study successfully synthesized PDA-Cur NPs, demonstrating its ability to stably load and release curcumin, with a loading percentage of 65.7% after 2 hours and 41.9% release in 8 hours (pH 6.0). Compared with single curcumin treatments, the photodegradation rates of PDA-Cur NPs in red and blue light treatments were reduced by 46% and 50%, respectively. Meanwhile, PDA-Cur NPs exhibited remarkable antitumor efficacy due to PDT and promote apoptosis in cancer cells, which both better than that of single curcumin treatments. Moreover, in MCF-7 tumor-bearing mice, the PDA-Cur NPs led to significant tumor growth inhibition effects, without causing evident systemic damage *in vivo*. The findings highlight the potential of PDA-Cur NPs as anticancer photosensitizer with greatly increased utilization of curcumin in PDT.

## Abstract 2

In this study, the PDA-Cur NPs has been successfully synthesized and proved to be an anticancer agent with better photostability and increased utilization of curcumin in PDT.

## Graphical abstract

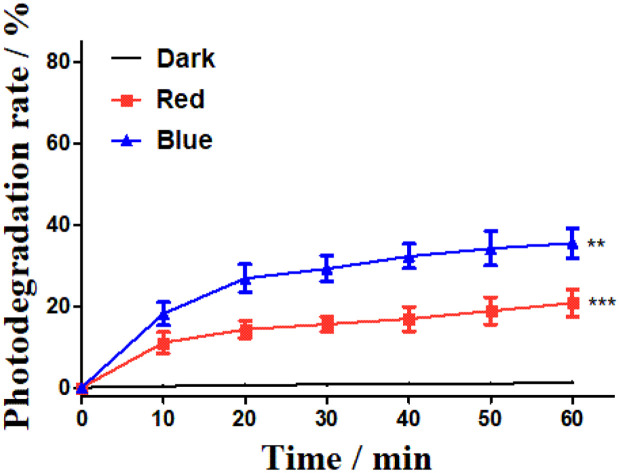

## 1 Introduction

Curcumin is currently one of the most widely used natural synthetic drugs in the world.^1–3^ It is a pigment molecule with photosensitive activity and has been proven to have effects such as in aging and senescence,^4,5^ food coloring,^6^ free radical scavenging,^10^ lipid-lowering,^11^ and as an anti-inflammatory,^7^ antioxidant,^8,9^ antibacterial,^12^ and anticancer agent.^13,14^ Curcumin mediated photodynamic technology has been applied in many industries, which is efficient and safety.^15,16^ As a photosensitive agent, curcumin generates reactive oxygen species (ROS) mainly composed of singlet oxygen species through the type II photosensitive reaction mechanism, thereby exerting its photodynamic effect.^17–19^ It was reported that the photodynamic effect of curcumin can effectively control microbial contamination in fresh agricultural and aquatic products.^20^ Furthermore, curcumin mediated photodynamic therapy (PDT) reaction has a significant killing effect on staphylococcus aureus and other bacteria.^21,22^ Importantly, the curcumin can also promote apoptosis of malignant cancer cells, inhibit their proliferation and metastasis.^23^ Curcumin can effectively inhibit the proliferation of cervical cancer cells due to PDT.^24^ Curcumin has been demonstrated to induce multiple cytotoxic effects in cancer cells including cell cycle arrest, apoptosis, autophagy, changes in gene expression, and disruption of molecular signaling.^25,26^ At present, the anti-tumor studies of curcumin are mostly confined to cell and animal experiments, which have shown that curcumin can inhibit cancer cell proliferation and apoptosis. Especially, curcumin-mediated photodynamic therapy has a typical inhibitory effect on human oral squamous cell cancer cells, kidney cancer cells and human epidermal cancer cells. In recent years, the most limiting problems of curcumin clinical application are high preparation and purification cost, low bioavailability, poor water solubility. Therefore, in order to improve the clinical application potential of curcumin, we must think about effective ways to solve these key problems.

The effect of photodynamic therapy is closely related to the properties of photosensitive drugs. Currently, a common bottleneck problem in the research and application of photosensitive drugs is their weak photostability, which is affected by special wavelength light to produce photodynamic effects and lead to photodegradation, thereby weakening the photodynamic effect of photosensitive drugs or shortening their use cycle.^27–29^ Although curcumin has been proven to have good anti-inflammatory, antioxidant, reducing blood sugar levels, lipid-lowering and autoimmune effects, there are still bottlenecks in its application,^30,31^ especially the photostability of curcumin, which leads to low bioavailability and seriously restricts the promotion and application of curcumin drug molecules.^32,33^ It is urgent to explore a more effective and suitable delivery method for curcumin to improve the effectiveness of the agent.

Dopamine is a catecholamine substance that can undergo oxidative self-polymerization in weakly alkaline aqueous solutions by controlling conditions, forming a type of polydopamine nanoparticles.^34,35^ Polydopamine nanoparticles has been proved to have good potential in clinical application and can become a kind of nanocarrier for clinical application. Polydopamine nanoparticles has been widely used in fields such as biomedicine with stable chemical properties, good biocompatibility and dispersibility. As a drug-carrier, polydopamine nanoparticles can load curcumin molecules through π–π interactions and electrostatic adsorption. The surface functional groups of polydopamine nanoparticles help to bind and load various drug molecules.^36–38^

To address problem above, in this study, we investigated the photostabilities of curcumin and its polydopamine-based nanoparticle after loading curcumin (PDA-Cur NPs) through *in vitro* and *in vivo* experiments to achieve better photostability and antitumor effects. Our findings demonstrate that the PDA-Cur NPs not only improves the photostability of curcumin, but also improves the application potential in anti-tumor therapy.

## 2 Experimental section

### 2.1 Materials and instruments

Experimental reagents curcumin (C_21_H_20_O_6_), dopamine hydrochloride, ammonia, anhydrous ethanol, *etc.* were mainly purchased from Alpha Chemical Co., LTD., Sigma-Aldrich (St Louis, USA) and Sinopsin Pharmaceutical Group Co., LTD. The human breast adenocarcinoma cell line MCF-7 was purchased from the Shanghai Institute of Cell Biology, Chinese Academy of Sciences, Shanghai, China. MCF-7 cells and Kunming mice (3–4 weeks old, 16–18 g, purchased from Shanghai SLAC Laboratory Animal Co. Ltd, Shanghai, China) were maintained and handled in accordance with the recommendations of the institutional animal care and use committee (IACUC). All experimental procedures conformed to ethical and moral requirements. MCF-7 cells were cultured in Dulbecco's modified Eagle's medium (DMEM) supplemented with 10% fetal calf serum (FCS) and 1% penicillin–streptomycin at 37 °C under humidified air with 5% CO_2_. MCF-7 cells were preserved in liquid nitrogen and passaged weekly through Kunming mice in the form of ascites to establish MCF-7 tumor-bearing mouse model. SP-752 UV spectrophotometer was purchased from Shanghai Spectral Instrument Co., LTD. Dynamic light scatterer Zetasizer Nano ZS 3600 from Malvern Instruments; Bison was purchased from Sigma Aldrich (Shanghai) Trading Co., LTD. YP6002 electronic balance was purchased from Jinan Oulebo Scientific Instrument Co., LTD. Blue light (425 ± 5 nm) and red light (620 ± 5 nm) were customized from Tehui Electronics (Hong Kong) Co., LTD.

### 2.2 Determination of curcumin standard curve

The maximum ultraviolet absorption wavelength of curcumin (425 nm) was selected as the detection wavelength. Weight 10 mg curcumin powder, dissolve in 100 mL 50% ethanol solution, dilute by multiple to prepare the curcumin working solution with 6.25 μg mL^−1^ (10% ethanol) and the UV absorption at 425 nm was measured. For determination of curcumin standard curve, curcumin working solution with different concentration gradient was configured, and the 425 nm UV absorption value of each sample group was measured by a Synergy 4 multi-mode microplate reader (BioTek Instruments, USA). With curcumin concentration as the horizontal coordinate and ultraviolet absorption value at 425 nm wavelength as the vertical coordinate, the standard curve was drawn and the *R*^2^ value was calculated.

### 2.3 Analysis of light stability of curcumin in dark

Add 10 mL of curcumin working solution (6.25 μg mL^−1^) to the beaker, wrap the beaker with tin foil, take 2 mL of curcumin working solution to measure the wavelength of 425 nm with a Synergy 4 multi-mode microplate reader and record it as the ultraviolet absorption value of 0 min. After measurement, pour it back into the beaker, leave it in the dark for 0.75 h, shake well. Take 2 mL of curcumin to measure the UV absorption value of 425 nm wavelength, and take photos of the sample cup for color comparison. After measurement, pour it back into the beaker and stand it for 10 minutes under dark conditions. Repeat the above steps to measure UV absorption at 425 nm wavelengths for 1.5, 3, 6, 12, and 24 h in dark conditions. With the light time as the horizontal coordinate and the photodegradation rate as the vertical coordinate, the line chart of the photodegradation rate of curcumin under dark conditions was drawn, and the color comparison analysis was carried out for each group of sample cups. Photodegradation rate = ((absorption measured at 0 min – absorption measured at *N* min))/(absorption measured at 0 min).

### 2.4 Measurement of curcumin photostability in red light

Take 10 mL of curcumin working liquid and add it to the beaker. Take 2 mL of curcumin working liquid and measure the wavelength of 425 nm with a spectrophotometer, record it as the ultraviolet absorption value of 0 min, and pour it back into the beaker under red light condition (as shown in Fig. S1A,† the red light is about 2 cm away from the mouth of the 50 mL beaker. Ensure that the whole solution plane is uniformly irradiated with red light) for 10 min (light dose is 12.5 J cm^−2^), shake well, take 2 mL curcumin and measure the ultraviolet absorption value of 425 nm wavelength with a spectrophotometer, and take photos of the sample cup for color comparison. After measurement, pour it back into the beaker and leave it for 10 min. The above steps were repeated to measure UV absorption values at 425 nm wavelengths for 20, 30, 40, 50 and 60 min in red light, and the photodegradation rate was recorded and calculated. With the light time as the horizontal coordinate and the photodegradation rate as the vertical coordinate, the line chart of the photodegradation rate of curcumin in the red light condition was drawn, and the color comparison analysis was carried out for each sample cup.

### 2.5 Measurement of curcumin photostability in blue light

Add 10 mL of curcumin working liquid to the beaker. Take 2 mL of curcumin working liquid and measure the wavelength of 425 nm with a spectrophotometer, record it as the ultraviolet absorption value of 0 min, and pour it back into the beaker under blue light condition (as shown in Fig. S1B,† the blue light is about 2 cm away from the mouth of the 50 mL beaker). Ensure uniform blue light irradiation of the whole solution for 10 min (light dose is 12.5 J cm^−2^), shake well, take 2 mL of curcumin working solution and measure the ultraviolet absorption value of 425 nm wavelength with a spectrophotometer, and take pictures of the sample cup for color comparison. After measurement, pour it back into the beaker and leave it for 10 min. The above steps were repeated to measure the UV absorption values at 425 nm wavelength under blue light conditions for 20, 30, 40, 50 and 60 min, respectively, and record and calculate the photodegradation rate according to the formula. With the light time as the horizontal coordinate and the photodegradation rate as the vertical coordinate, the line chart of the photodegradation rate of curcumin under blue light condition was drawn, and the color comparison analysis was carried out for each sample cup.

### 2.6 Synthesis of polydopamine nanocarriers

Polydopamine nanocarriers are formed by the auto-polymerization of dopamine hydrochloride under appropriate conditions. Add 10 mL distilled water and 10 mL anhydrous ethanol into the same beaker and place them in a magnetic stirrer for stirring. During the stirring process, absorb 0.5 mL ammonia water and add it into the beaker, and continue to stir at constant temperature. Weight 100 mg dopamine hydrochloride powder, absorb 1 mL distilled water to dissolve it fully, add it to the beaker as soon as possible, keep the system stirred at a constant speed for 12 h, then centrifuge the beaker solution separately (12 000 rpm, 2 min), carefully remove the supernatant with a pipette, and repeat the operation for 5 to 7 times. After centrifugation, the supernatant was water transparent, indicating that the washing and purification of the polydopamine nanomaterial had been completed. After centrifugation, the precipitated polydopamine nanoparticles were used for subsequent curcumin load characterization and detection. The size distribution and polydispersity index of polydopamine nanoparticles were determined by dynamic light scattering (DLS) measurements (Nano ZS90, Malvern Instruments, UK).

### 2.7 Synthesis of polydopamine-curcumin nanoparticles (PDA-Cur NPs)

The UV absorption spectrum of polydopamine has a broad spectrum, with absorption in the range of 400–800 nm, but no clear absorption peak, while curcumin has a maximum UV absorption peak at 425 nm. Therefore, the experiment was designed to calculate the loading effect of polydopamine nanocarriers on curcumin by measuring the change of UV absorption value at 425 nm wavelength of the loading system. Three beakers were taken, and 30 mL of curcumin working liquid (8 μg mL^−1^) was measured in two beakers, and 30 mL of distilled water was measured in the other beaker as the control group. The beakers were placed in a magnetic stirrer and stirred at a constant speed. Add 10 mg of the above-mentioned prepared polydopamine nanoparticles into three beakers and stir them fully and continuously in the curcumin working liquid system. Collect 4 mL of each experimental group every 1 h. After centrifugation, the supernatant is tested for 425 nm absorption value. The loading and loading percentage of polydopamine nanocarriers on ginger were calculated until there was no significant change in light absorption value measured for 3 consecutive times (loading plateau period). After that, the prepared PDA-Cur NPs were purified, the unstable load of curcumin was fully washed away for analysis, and then characterized and used for backup. For curcumin releasing measurement, PDA-Cur NPs was dialyzed in PBS buffer at pH 6.0 in dark and kept in a 37 °C water bath. At different time points (1, 2, 4, 8 and 16 h), curcumin released from PDA-Cur NPs was collected. The size distribution and polydispersity index of PDA-Cur NPs were determined by dynamic light scattering (DLS) measurements (Nano ZS90, Malvern Instruments, UK).

### 2.8 Stability and photodegradation of PDA-Cur NPs

To study the stability of PDA-Cur NPs, we dispersed them in saline and kept standing at room temperature and determined the size distribution by DLS on day 0, 4, 8. Loading curcumin with polydopamine nanocarriers with its good biocompatibility is expected to slow down the photodegradation. The photostability of PDA-Cur NPs in dark, red and blue light treatments were studied to investigated the slowing effect of composite nanoparticles on their photodegradation. The control group (dark group), red group and blue group of PDA-Cur NPs were set. Dark, blue and red light treatments were carried out respectively to measure the photodegradation rate in the three groups. The three components were evenly dispersed in the aqueous solution, the light was continuously illuminating for 60 min, and 2 mL solution was taken every 10 min to measure the ultraviolet absorption value of 425 nm wavelength with a spectrophotometer. After the measurement was completed, the solution was poured back into the beaker, and the above steps were repeated to measure 20, 30, 40, 50 and 60 min respectively in the three conditions. UV absorption values at 425 nm wavelength in each time were recorded and the photodegradation rate was calculated according to the formula. With light time as horizontal coordinate and photodegradation rate as vertical coordinate, the photodegradation rate of PDA-Cur NPs under dark, red and blue light conditions was plotted.

### 2.9 Tumor cytotoxicity of PDA-Cur NPs

The survival of MCF-7 cells upon treatment with PDA-Cur NPs was evaluated using standard methyl thiazolylte-trazolium (MTT) assays, which assess cell viability based on the reduction of yellow MTT to insoluble purple formazan. MCF-7 cells at a density of 5 × 104 cells per mL were collected and plated in 96-multiwell plates with culture medium (200 μL per well). The MCF-7 cells were divided into three groups, including control, curcumin (dark and lighting) and PDA-Cur NPs (dark and lighting) groups. The cells of three groups were incubated with medium, curcumin and PDA-Cur NPs for 2 hours respectively. The cells of lighting treat groups followed by a 2 minute illumination at doses of 5 J cm^−2^ using a planar LED light source (425 nm). Dark toxicity experiments on MCF-7 cells were conducted simultaneous. After 24 hours incubation, 100 μL MTT solution (0.5 mg mL^−1^) in DMEM was added to the cell plate and continued to culture for 3 hours. The insoluble purple formic acid product was dissolved in 100 μL dimethyl sulfoxide (DMSO) to obtain a colored solution. Its absorbance was measured at 570 nm using a Synergy 4 multi-mode micro-plate reader. Each experiment was repeated three times with three replicates per con-centration.

### 2.10 Apoptosis and microscopy images of MCF-7 cells by PDA-Cur NPs

Apoptosis in MCF-7 cells induced by curcumin and PDA-Cur NPs with light treatments were detected and analyzed using Annexin V-FITC Apoptosis Detection Kits (Beyotime Biotechnology, China). Annexin V conjugated with the fluorochrome FITC binds to membrane phospholipid phosphatidylserine, serving as a sensitive probe for flow cytometric analysis of cells undergoing apoptosis. The MCF-7 cells were plated at a density of 5 × 10^4^ cells per mL (2 mL per well) in four 6-well plates. After 12 hours of incubation at 37 °C for cell adhesion, the cells were treated with curcumin or PDA-Cur NPs for 2 hours. According to curcumin standard curve and loading percentage results, curcumin content of curcumin group and PDA-Cur NPs was same. After careful washing with PBS, the all cells were subjected to a 2 minute illumination (425 nm LED light source). Cells without any drugs served as a control. Then, the apoptosis rate of MCF-7 cells after treatments were measured. For microscopy image assay, the MCF-7 cells were incubated with PDA-Cur NPs and further irradiated by LED light source (425 nm). The microscopy imaging of cells before and after treated with PDA-Cur NPs were imaged by confocal laser scanning microscopy (Olympus FluoView™ FV1000, Japan). MCF-7 cells were planted into 6-well plate at a density of 5 × 10^4^ per mL culture medium in a volume of 2 mL per well and incubated overnight. Then the cells of treatment group were incubated with PDA-Cur NPs for 30 min respectively. The plates were washed thrice with PBS carefully before a 2 minute illumination (425 nm LED light source). The MCF-7 cells without any treatment as control.

### 2.11 Establishment of MCF-7 tumor-bearing mouse model

Kunming mice (3–4 weeks old, 16–18 g) were maintained and handled in accordance with the recommendations of the institutional animal care and use committee (IACUC). The all mice were allowed free access to food and water throughout the experiments. To establish the mouse MCF-7 tumor model, ascites containing highly active MCF-7 cells were harvested from the peritoneal cavity from the tumor-bearing mouse after inoculation many days, diluted with sterilized saline to adjusted the concentration at a density of 1.0 × 10^7^ cells per mL, and 0.2 mL aliquots were subcutaneously inoculated into the right side of the mice back. The experiments started when the tumor length (the longest diameter of the tumor) and width (diameter perpendicular to the length) reached 5–7 mm and 4–6 mm, respectively.

### 2.12 *In vivo* antitumor effect of PDA-Cur NPs

The antitumor activity of PDA-Cur NPs against MCF-7 tumor-bearing mice models implanted in Kunming mice was evaluated by body weight and growth inhibition (relative tumor volume) analysis. Typically, MCF-7 tumor-bearing mice (established as above) were randomly divided into five groups (8 mice per group), including control, curcumin in dark (CUR Dark), curcumin in light (CUR Lighting), PDA-Cur NPs in dark (PDA-CUR Dark) and PDA-Cur NPs in light (PDA-CUR Lighting). The *in vivo* assay was started with the equivalent average starting tumor size (∼35 mm^3^) and body weight (∼21.65 g). The mice of curcumin and PDA-Cur NPs treatment group were administered curcumin or PDA-Cur NPs *via* the caudal vein while the control group was injected intravenously with equivalent volume of 0.9% saline. 8 h later, MCF-7 tumors in lighting groups were treated with a 450 nm light illumination (LumaCare non-coherent light source, Luma Care Medical Group, Newport Beach, CA, USA) for 10 min to get a total light fluence of 100 J cm^−2^. The body weights of mice were monitored daily and the tumor sizes were calculated with a caliper throughout the experiment using the ellipsoid volume formula (*W*^2^ × *L*) × π/6, where *W* means tumor width and *L* means tumor length. Upon completion of the 8 day PDT treatment, mice were sacrificed. The PDT efficacies against the MCF-7 tumor in mice were evaluated by a tumor growth-inhibition and tumor weight analysis.

### 2.13 Statistical analysis

All data represent group means and standard errors of the mean (SEM). The experimental data was analyzed by two-way analyses of variance (ANOVA). Differences at the 95% confidence level (*p* < 0.05) were considered statistically significant.

## 3 Results and discussion

### 3.1 Standard curve of curcumin

**Fig. 1:**
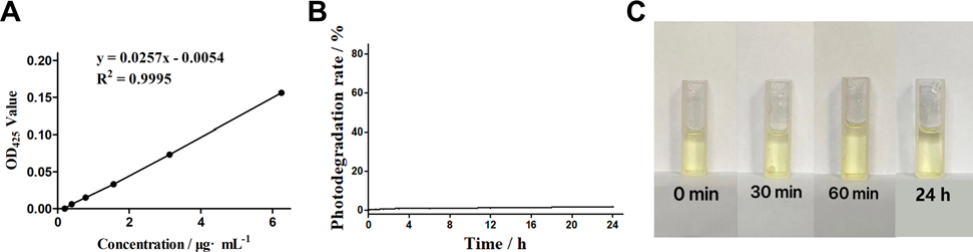
Standard curve of curcumin and light stability of curcumin in dark conditions. (A) Standard curve of curcumin. (B) Curcumin photodegradation rate. (C) Color change of curcumin in dark.

The determination of curcumin standard curve is helpful to analyze the relationship between curcumin concentration and absorbance, evaluate the UV absorption stability of curcumin, and provide a basis for the subsequent study of the influence of curcumin light stability. The results of curcumin standard curve are shown in Fig. 1A. The results show that curcumin has a good linear relationship with its corresponding 425 nm UV absorbance value within the measured concentration range (0.19–6.25 μg mL^−1^), and the linear equation is *y* = 0.0257*x* − 0.0054 (*R*^2^ = 0.9995). It was proved that the selected curcumin had good quality with stable UV absorption, which could be used for the experimental study of its light stability.

### 3.2 Photo-stability of curcumin in dark

Curcumin as a photosensitive agent is stored in a light-free environment to maintain its stability. The investigation of the curcumin photodegradation in dark conditions is helpful to understand its photostability in dark, and to compare with the subsequent red and blue experimental groups. Fig. 1B shows the photodegradation rate of curcumin after standing in the dark for 0–24 hours, suggesting that curcumin has almost no photodegradation phenomenon (all of which are less than 2.5%) within 0–24 h. Such results proves that curcumin has good photostability in dark and is expected to maintain good photodynamic efficacy within the measured time range. The light stability of curcumin under dark conditions is also reflected in the color change of sample cups in each time group (Fig. 1C). There is no significant change in the color of sample solution in each time group where curcumin is placed in a light-free static environment for 0–24 h, demonstrating that curcumin has low photodegradation rate and good photostability in dark conditions.

### 3.3 Analysis of photo-stability of curcumin in red light

**Fig. 2:**
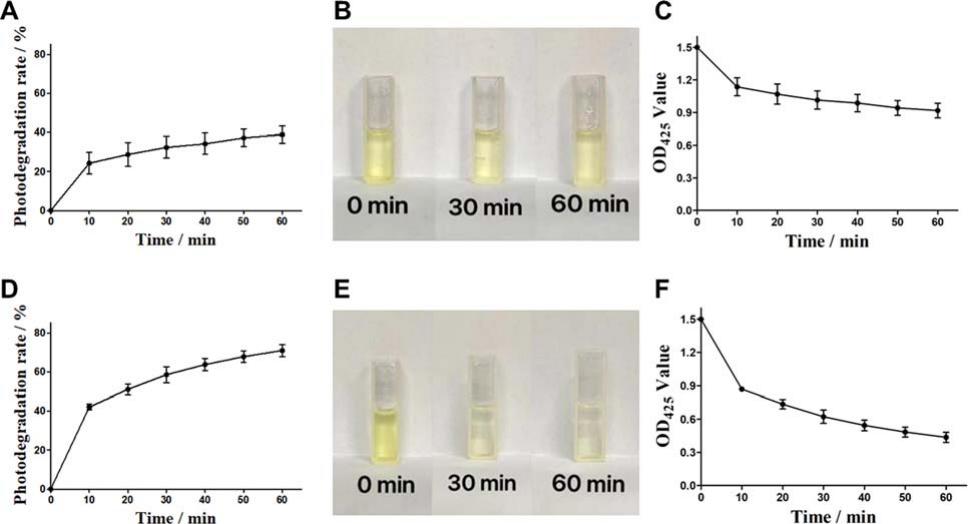
Light stability of curcumin with red and blue light. (A) Curcumin photodegradation rate in red light. (B) Color change of curcumin in red light. (C) OD_425_ change of curcumin in red light. (D) Curcumin photodegradation rate in blue light. (E) Color change of curcumin in blue light. (F) OD_425_ change of curcumin in blue light.

Red light with the characteristics of long wavelength, deep penetration and can act on thick tissues has become one of the most common light sources in daily life. It was reported that red light is conducive to increasing curcumin content in curcumin roots.^24^ Therefore, to analysis the effect of red light on the curcumin is contributed to measure its tolerance and light stability to red light during its photodynamic role. Curcumin was exposed to red light for 10 min each time for 6 consecutive times. As shown in Fig. 2A, curcumin exhibit obvious photodegradation under red light, showing rapid photodegradation under red light in the first 10 min, with a degradation rate of 24.3%. The photodegradation under red light in the next 20 to 60 min was positively correlated with light time (dose). However, the growth rate of photodegradation decreased, and the photodegradation rate of 60 min red light group was 38.8%, which tended to be stable. In Fig. 2B, the color changes of curcumin samples in the red light group after 30 or 60 min of illumination, indicating that the color of curcumin changes from bright yellow to light yellow. The Fig. 2C showed the similar trend as the OD_425_ measurements. With the increase of light time, the OD_425_ absorption value of curcumin gradually decreased and reached 0.917 at 60 min. In summary, the results demonstrate that red light significantly affect the photostability of curcumin.

### 3.4 Analysis of photo-stability of curcumin in blue light

It was reported that the curcumin was able to absorb blue light in the wavelength range of 400–500 nm, with a maximum absorption band at 425 nm. Therefore, blue light with a wavelength 425 nm is commonly used as the excitation light source of curcumin, to realize the photosensitive activity of curcumin. In our study, the photodegradation rate of curcumin in blue light was investigated to analyze its photostability and utilization rate of curcumin. Curcumin was exposed to blue light for 10 min each time (light dose was 12.5 J cm^−2^) for 6 consecutive times. The results were shown in Fig. 2D, showing a strong photodegradation rate of curcumin in blue light. In the first 10 min, the photodegradation rate of curcumin reached 42.1%, which was 1.7 times that of the red light dose group. With the increase of blue light time (dose), the degree of photodegradation of curcumin still showed a steady increasing trend. The photodegradation rate of 60 min blue light group was 70.9%, which was 1.8 times that of the red light dose group. Such significant photodegradation effect of blue light on curcumin were also reflected in the color changes and OD_425_ measurements with different time (Fig. 2D and F). It was obviously observed that the color of the samples changed rapidly with continuous blue illumination, showing rapid light fading, from bright yellow to weak yellow. The OD_425_ measurements also exhibited a rapid decline trend during 60 min blue illumination (0.436, compared with 0.917 in red light group). The experimental results showed that curcumin was significantly affected by blue light and showed stronger photodegradation than red light.

### 3.5 Synthesis of polydopamine nanocarriers

**Fig. 3:**
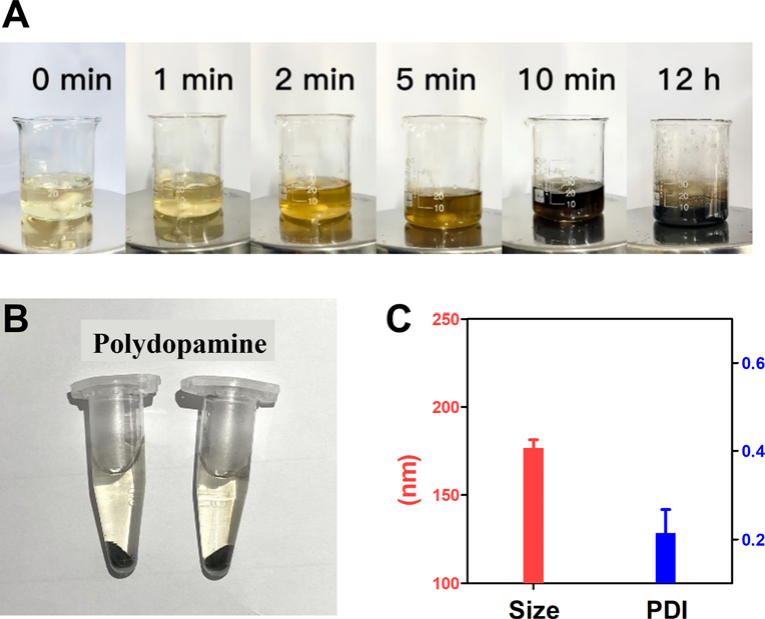
Synthesis of polydopamine nanocarriers. (A) Synthesis process of polydopamine. (B) Sample preparation. (C) Size distribution and PDI analysis of polydopamine.

The synthetic process of polydopamine nanocarriers showing significant color changes, which can be used to determine whether the self-polymerization of dopamine hydrochloride is smooth. The process of polydopamine synthesis can be seen from Fig. 3A, the color of the solution rapidly changes from light yellow to dark brown at the 0–5 min stage, and the solution tends to be black at the 10th min of agitation, and continues to maintain dark black in the subsequent 12 h reaction. After reaction, the obtained polydopamine nanomaterial was purified to obtain black particle precipitation (Fig. 3B), and its particle size was determined. The results showed that the polydispersity index (PDI) of the prepared polydopamine nanoparticles was 0.215, and the average particle size was 176.7 nm (Fig. 3C), which proved that the polydopamine nanoparticles had uniform particle size with well dispersion coefficient.

### 3.6 Synthesis of PDA-Cur NPs

**Fig. 4:**
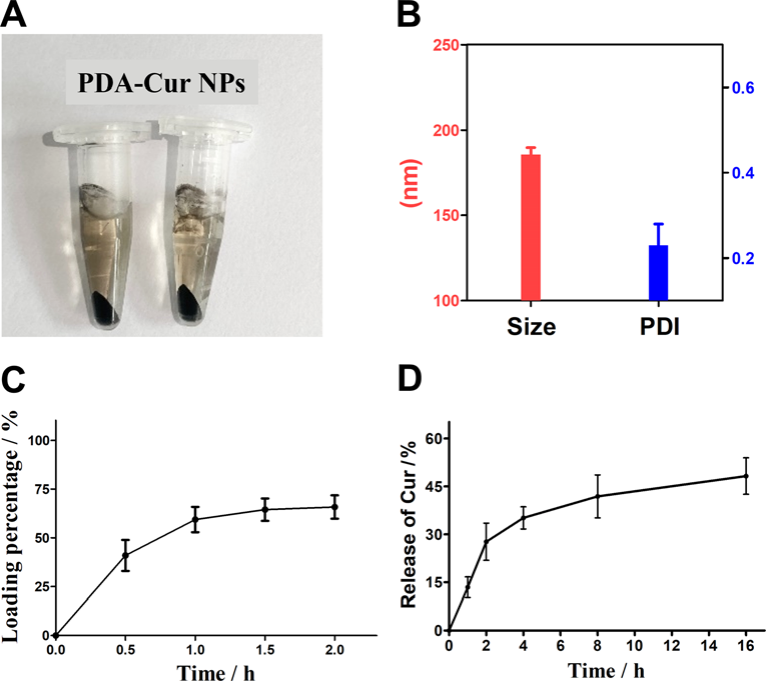
Synthesis and characterization of PDA-Cur NPs. (A) Preparation sample of PDA-Cur NPs. (B) Size distribution and PDI analysis of PDA-Cur NPs. (C) Dynamic loading of curcumin in polydopamine. (D) Release of curcumin form PDA-Cur NPs *versus* time in PBS at pH 6.0.

Polydopamine has been widely used in many fields due to its good biocompatibility to provide more reliable stability for loaded drugs. The polydopamine nanoparticles could loading with curcumin molecules by π–π interaction and electrostatic adsorption. In this study, PDA-Cur Np was prepared and purified (Fig. 4A). There was no significant difference between PDA-Cur and polydopamine nanoparticles in appearance. Meanwhile, particle size comparative analysis results before and after curcumin loading with polydopamine also proved that curcumin was successfully loaded on polydopamine nanoparticles. The average particle size of PDA-Cur NPs was 185.7 nm (Fig. 4B), which increased by 5.1% compared to the polydopamine nanoparticles. The PDI of the PDA-Cur NPs was 0.229, indicating that the composite nanoparticle possess similar uniform and good dispersion similar to polydopamine. As shown in Fig. 4C, the loading percentage of curcumin increases rapidly with the loading time in the first 1 h, and then reach loading plateau period in 2 hours, demonstrating that the curcumin can be stably loaded on the PDA-Cur NPs, which shown a final loading percentage of curcumin was about 65.7%. Furthermore, we also investigated the release behavior of curcumin in PBS buffer (pH 6.0). The result was shown in Fig. 4D, which indicated that curcumin was able to have an observably release within 2 hours (27.7%). Curcumin was rapidly released within 4–8 hours and reaches a drug release plateau (8 hours, 41.9%), mainly owing to the protonation of the amino group in the nanoparticles, thus facilitating drug release under acidic pH.

### 3.7 Stability and photodegradation of PDA-Cur NPs

**Fig. 5:**
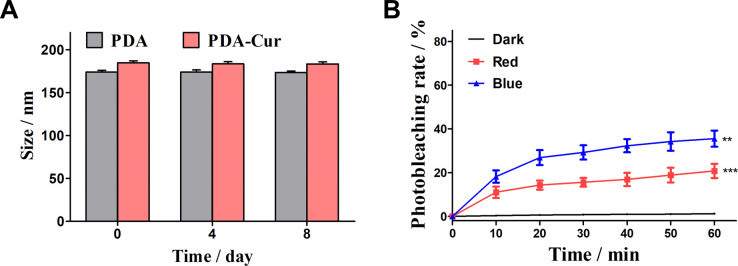
Stability and photodegradation of PDA-Cur NPs. (A) Analysis of stability of PDA-Cur NPs in 8 days. (B) Photodegradation rate of PDA-Cur NPs with dark, red and blue treatment. The values were represented as mean ± SEM, ***p < 0.001, **p < 0.01, *p < 0.05 *vs.* the control group.

In this study, synthesis of PDA-Cur NPs with good biocompatibility is expected to slow down the photodegradation of curcumin. The stability in 8 days and photodegradation of PDA-Cur NPs in dark, red and blue light were investigated to explore the slowing effect of composite nanoparticles on their photodegradation. As shown in Fig. 5A, the PDA-Cur NPs exhibited high stability in 8 days without significant size change. Notably, PDA-Cur NPs shown the well photostability in dark conditions, and almost no significant photodegradation occurs within the measured time range (Fig. 5B). Although the photodegradation of PDA-Cur NPs occurred under continuous exposure to red and blue light, the photodegradation degree was significantly lower than that of the no-loading group (single curcumin). In the red light group, the degree of photodegradation produced by PDA-Cur NPs gradually increased with the increase of time, the photodegradation rate was 11.1% at 10 min of light (12.5 J cm^−2^), and then the increase of photodegradation slowed down, reaching 20.8% at 60 min. It was 46% lower than the single curcumin red light group (38.8%) under the same condition. For the blue light group, PDA-Cur NPs also showed the similar trend, which could significantly reduce the photodegradation of curcumin, and the photodegradation rate was 18.2% after 10 min illumination (12.5 J cm^−2^) and 35.5% after 60 min illumination. It is about 50% of the single curcumin blue light group (70.9%) under the same conditions, demonstrating that PDA-Cur NPs can reduce the degree of photodegradation of curcumin by nano-loading form, which is expected to improve the photostability of curcumin, and provide help for achieving better photodynamic effect.

### 3.8 Tumor cytotoxicity and apoptosis of PDA-Cur NPs

**Fig. 6:**
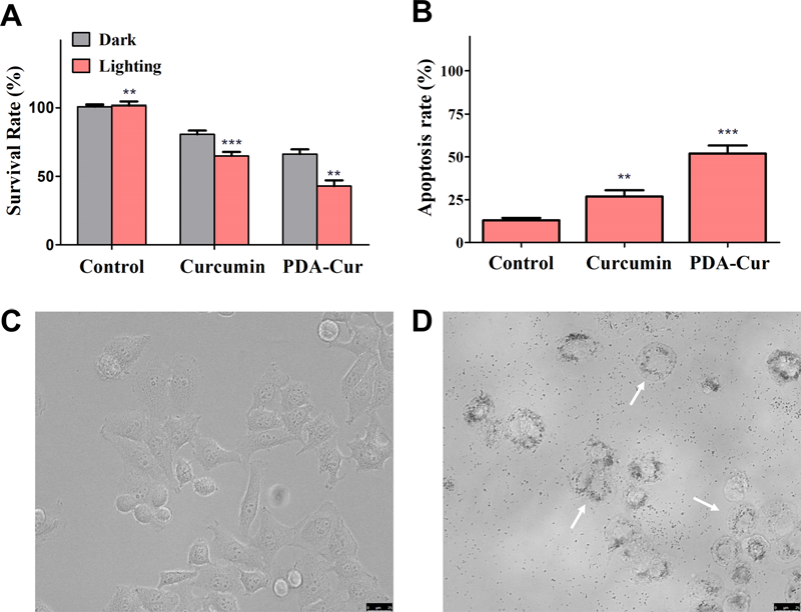
*In vitro* antitumor effects of PDA-Cur NPs. (A) Survival rates of curcumin and PDA-Cur NPs to MCF-7 cells. (B) Apoptosis rates of curcumin and PDA-Cur NPs to MCF-7 cells. (C) Microscopy images of MCF-7 cells without any treatment. (D) Microscopy images of MCF-7 cells incubated with PDA-Cur NPs. The values were represented as mean ± SEM, ***p < 0.001, **p < 0.01, *p < 0.05 *vs.* the control group. Scale bars represent 25 μm.

The antitumor effects *in vitro* of PDA-Cur NPs has been investigated in MCF-7 cells. The cytotoxicity of PDA-Cur NPs against MCF-7 cells was evaluated using the standard MTT assay. MCF-7 cells were treated with curcumin and PDA-Cur NPs respectively. To investigate the PDT effects of curcumin, we further setting a group with or without light treatment to assess the potential PDT by curcumin. The phototoxicity of PDA-Cur NPs on MCF-7 cells was shown in Fig. 6A as survival rates forms, where the survival rate of MCF-7 cells significantly decreased after curcumin or PDA-Cur NPs treatments. In single curcumin treatments, the survival rates of MCF-7 cells were 80.6% and 64.9% with or without illumination respectively. As a contrast, the survival rates of MCF-7 cells in PDA-Cur NPs treatments were drop to 66.3% and 42.9% compare to single curcumin treatments, demonstrating that the PDA-loading of curcumin observably improve its PDT efficiency. A similar result was also observed in apoptosis measurements, the PDA-loading of curcumin (PDA-Cur NPs) leading to greater apoptosis rate (Fig. 6B). Notably, PDA-Cur NPs treatments shown a significant apoptosis (52.0%), which was 1.94- and 4-fold higher than that of single curcumin (26.8%) and control group (13.0%), respectively. Furthermore, MCF-7 cells treated with or without PDA-Cur NPs were used to investigate the microscopy imaging and apoptosis. Fig. 6C shows the normal form of MCF-7 cells without PDA-Cur NPs treatment. Significantly, the MCF-7 cells after treated with PDA-Cur NPs and PDT treatments shown the state of apoptosis (Fig. 6D), especially cell vacuolation and broken, demonstrating the PDA-Cur NPs and PDT effects on MCF-7 cells. These findings further suggest that the PDA-Cur NPs treatments perhaps exhibit the synergistic PDT and nano-medicine effects to achieve better antitumor efficacy.

### 3.9 *In vivo* antitumor effect of PDA-Cur NPs

**Fig. 7:**
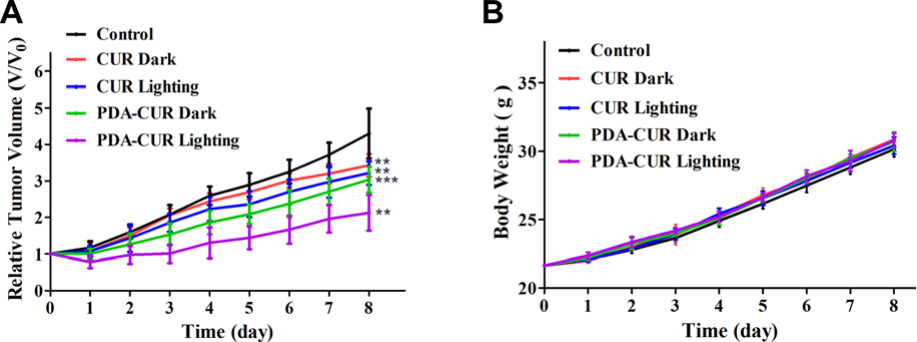
Body weight (A) and relative tumor volume (B) of curcumin and PDA-Cur NPs to MCF-7 tumor-bearing mice. The tumor volumes were normalized to their initial sizes (*V*/*V*_0_). The values were represented as mean ± SEM, ***p < 0.001, **p < 0.01, *p < 0.05 *vs.* the control group.

The antitumor activity of PDA-Cur NPs against MCF-7 tumor-bearing mice models implanted in Kunming mice was evaluated by body weight and growth inhibition (relative tumor volume) analysis. Typically, MCF-7 tumor-bearing mice (established as above) were randomly divided into five groups (8 mice per group), including control, curcumin in dark (CUR Dark), curcumin in light (CUR Lighting), PDA-Cur NPs in dark (PDA-CUR Dark) and PDA-Cur NPs in light (PDA-CUR Lighting). As shown in Fig. 7A, all the curcumin and PDA-Cur NPs groups exhibited significant reduction in tumor growth rates compared to the control group after 8 days of treatment. Especially, on the 8th day, the relative tumor volume of the PDA-CUR lighting group (2.12), PDA-CUR dark group (3.04), CUR lighting group (3.22) and CUR dark group (3.43) exhibited a 2.03-fold, 1.41-fold, 1.34-fold and 1.25-fold reduction compared to the saline group (4.30), respectively. It should be noted that the PDA-CUR lighting group shown better inhibition ratios compared to the CUR lighting group (1.52-fold reduction) and PDA-CUR dark group (1.43-fold reduction), confirming the significant advantage and synergistic effect of PDT and nano-medicine effects compared to single curcumin therapy. Meanwhile, the mice in each group showed increased body weights after treatment (Fig. 7B), indicating that the therapy mediated by PDA-Cur NPs exerted no significant side effects *in vivo*.

## 4 Conclusions

In summary, to enhance the photostability of photosensitizer curcumin, a polydopamine-based nanoparticle (PDA-Cur NPs) loaded with curcumin was synthesized and investigated *in vitro* and *in vivo* to achieve better photostability and antitumor effects. Our results demonstrated that curcumin has good photostability in dark, but with significant photodegradation rates in both red and blue light conditions. Blue light has a faster effect on the photodegradation of curcumin, with a degradation rate of 42.1% after 10 minutes of light exposure, which is about 1.7 times that of the red light group. Our study successfully synthesized PDA-Cur NPs, demonstrating their ability to stably load and release curcumin, with a loading percentage of 65.7% after 2 hours and 41.9% release in 8 hours (pH 6.0). Compared with single curcumin, the photodegradation rates of PDA-Cur NPs in red and blue were reduced by 46% and 50%, respectively. Meanwhile, PDA-Cur NPs exhibited remarkable antitumor efficacy due to PDT and promote apoptosis in MCF-7 cells. Moreover, in MCF-7 tumor-bearing mice, the PDA-Cur NPs led to significant tumor growth inhibition effects, without causing evident systemic damage *in vivo*. Our study highlights the potential of PDA-Cur NPs as anticancer photosensitizer with better utilization of curcumin in PDT.

## Conflicts of interest

The authors have declared that no competing interest exists.

## Supplementary Material

RA-014-D4RA01246A-s001
