# Supplementary material for: Photostabilities and anti-tumor effects of curcumin and curcumin-loaded polydopamine nanoparticles
Source: RSC Adv. 2024 Apr 25;14(20):13694–702. doi: 10.1039/d4ra01246a (PMC11044124; doi:10.1039/d4ra01246a)
Supplement: RA-014-D4RA01246A-s001 [file RA-014-D4RA01246A-s001.pdf]

Electronic Supplementary Material (ESI) for RSC Advances.
This journal is © The Royal Society of Chemistry 2024

Supporting Information

Photostabilities and anti-tumor effects of curcumin and curcumin-loaded polydopamine nanoparticle

Shufeng Yan,*[a] Xiaoyun Liao,[a] Qi Xiao,[a] Qingqing Huang[a] and Xiaochen Huang[b]

[a] Medical Plant Exploitation and Utilization Engineering Research Center, Sanming University, Sanming, Fujian 365004, China.

[b] State Key Laboratory of Structural Chemistry, Fujian Institute of Research on the Structure of Matter, Chinese Academy of Sciences, Fuzhou, Fujian 350002, China.

E-mail: Shufeng Yan, ysfready@fjsmu.edu.cn

Contents

1. **Figure S1**. Illumination of red (A) and blue (B) light on curcumin.

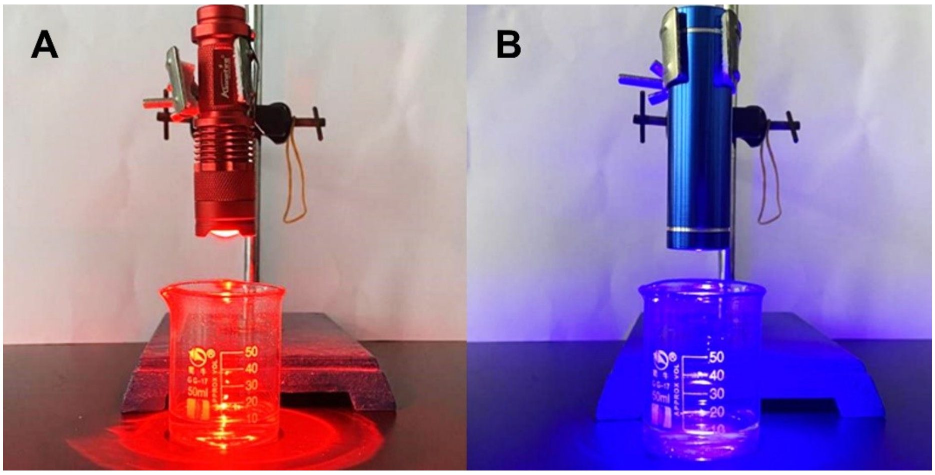

Figure S1 Illumination of red (A) and blue (B) light on curcumin
